# Why do Canadians host refugees? A sequential explanatory mixed-methods study protocol

**DOI:** 10.1371/journal.pone.0353551

**Published:** 2026-07-13

**Authors:** Areej Al-Hamad, Yasin Yasin, Kateryna Metersky, Aryan Karimi, Sepali Guruge, Maher El-Masri, Riham Al-Saadi

**Affiliations:** 1 Daphne Cockwell School of Nursing, Toronto Metropolitan University, Toronto, Ontario, Canada; 2 Faculty of Nursing and Health Sciences, University of New Brunswick, Fredericton, New Brunswick, Canada; 3 Department of Sociology, University of British Columbia, Vancouver, British Columbia, Canada; 4 School of Social Work, University of Windsor, Windsor, Ontario, Canada; Public Library of Science, UNITED STATES OF AMERICA

## Abstract

Refugee homestay hosting, in which private citizens or permanent residents provide temporary accommodation and informal support to refugees and displaced persons, is increasingly used as a community-based response to housing insecurity, social isolation, and settlement barriers. Although prior scholarship has examined refugee experiences within hosting arrangements, comparatively little is known about the motivations, readiness, attitudes, empathy, satisfaction, and advocacy behaviours of hosts, or about the organizational supports required to sustain hosting in Canada. The study is informed by hospitality studies and collective action perspectives. This protocol describes a 36-month sequential explanatory mixed-methods study of three phases designed to examine why Canadians host refugees and how refugee homestay hosting can be strengthened through evidence-informed and co-designed strategies. Phase 1 will include the administration of a national survey to current and former Canadian refugee homestay hosts to examine relationships among hospitableness, attitudes toward refugees, empathy, readiness to assist, satisfaction, and advocacy for refugee hosting. Quantitative data will be analyzed using descriptive and inferential statistics. Open-ended survey responses will be thematically analyzed. Phase 2 will use qualitative descriptive methods to conduct semi-structured interviews with 20–25 Canadian hosts and service providers to explore motivations, practices, needs, challenges, trauma- and gender-responsive considerations, and recommendations for service and policy improvement. Phase 3 will use group concept mapping and co-design sessions with 20–25 hosts and service providers to prioritize needs and develop multi-level action-oriented recommendations, including a host support toolkit, service provider guidelines, and policy strategies. Findings are expected to inform refugee-serving organizations hosting practices and programs to sustain safe, inclusive, and effective refugee homestay hosting in Canada.

## Introduction

Canada has experienced sustained refugee and displaced population arrivals, creating urgent needs for temporary accommodation, settlement support, and socially inclusive pathways into communities [1–3]. Refugee and displaced population often face barriers to securing safe and affordable housing, including financial constraints, limited system knowledge, and social isolation [2]. In response, humanitarian, community, and refugee-serving organizations have implemented homestay hosting initiatives in which local residents provide accommodation in their homes for a defined period [4–10]. These arrangements may support social connection, language acquisition, navigation of services, employment support, and everyday familiarity with the receiving communities [5,11–15].

Homestay hosting is increasingly positioned as both a short-term shelter response and a relational settlement model, particularly for refugee women and families, which often includes their children or dependent parents [8,10,13,16,17]. Similar approaches have been adopted in several countries such as Australia, the United Kingdom, France, Belgium and Finland [8,18–20,21–27]. Although hosting can generate social inclusion, belonging, and practical support, it also involves complex domestic, cultural, emotional, and power-laden dynamics [5,8,9,21,22,27]. Hosts may be motivated by humanitarian values, moral responsibility, political solidarity, faith or community commitments, dissatisfaction with host country responses, and/or a desire to counter exclusionary narratives about refugees [18,21–26].

Existing research has largely emphasized refugee perspectives, with less attention to the host as an active participant in the production of care, welcome, and advocacy [5,28,29]. A previous study with displaced Ukrainian women and their Canadian hosts in Toronto highlighted the potential of homestay hosting to create a sense of “home away from home,” while also showing that hosting requires clearer expectations, preparation, boundaries, and support mechanisms [12,30]. These findings underscore the need to examine how hosts become involved and enacted hospitableness, how their attitudes and empathy develop, and what conditions influence satisfaction and willingness to host refugees.

Hospitableness, defined as offering hospitality generously and without expectation of repayment [31], may be a key driver of positive contact between hosts and refugees [32]. Contact with refugees can improve attitudes and reduce prejudice, while empathy can foster responsibility, readiness to help, and advocacy behaviours [29,33–39]. Satisfaction is also important because positive hosting experiences may influence intentions to host again, recommend hosting to others, and promote hosting within community networks [40,41]. However, the pathways linking hospitableness, attitudes, empathy, readiness to assist, satisfaction, and advocacy remain underexplored in the Canadian refugee homestay context. This study responds to this gap by combining national survey data, qualitative interviews, and group concept mapping co-design to generate practice- and policy-relevant evidence.

## Study objectives

The overall aim of this study is to explore why Canadians host refugees and to co-design recommendations and strategies to strengthen and sustain refugee homestay hosting in Canada. The study has three objectives:

1. To examine the mediating effects of attitudes toward refugees, empathy, satisfaction, and readiness to assist on the relationship between hospitableness and advocacy for refugee hosting among Canadian hosts.
2. To explore the experiences of Canadian hosts and service providers with refugee homestay hosting, including perceptions, readiness, motivations, challenges, satisfaction, and supports required to sustain engagement.
3. To co-design multi-level, action-oriented recommendations and strategies with hosts and service providers to strengthen and sustain Canadian refugee homestay hosting practices.

### Theoretical framework

This study is informed by an integrated framework that brings together hospitality studies and collective action perspectives [42]. Hospitality studies provide a lens for examining the relational, symbolic, ethical, and power-laden dimensions of host-guest encounters [43–50]. In refugee hosting, hospitality involves more than the provision of shelter; it includes ways societies and households use to regulate openness, belonging, obligation, and boundaries [46–49]. Derrida’s theory of hospitality highlights the paradox between the ethical ideal of unconditional welcome and the practical limits imposed by the host’s authority over the home, including eligibility, behavioural expectations, duration of stay, and household rules [47,50]. This lens is useful for examining the asymmetry, ambivalence, and negotiated boundaries embedded in homestay hosting. At the same time, refugee homestay hosting cannot be understood only as a private household decision. Hosting is embedded in collective action, community mobilization, organizational coordination, and broader refugee support infrastructures [51–53]. Collective action perspectives help explain how social norms around helping others are constructed, how communities foster cooperation and altruistic action, and how hosts become advocates for refugee support [51–53]. Integrating these perspectives allows the study to examine micro-level domestic practices of welcome alongside macro-level systems, services, policies, and advocacy processes that shape the sustainability of hosting.

## Materials and methods

### Study design

This 36-month sequential explanatory mixed-methods study of 3 phases begins with a quantitative survey of Canadian hosts (phase 1), followed by qualitative exploration with hosts and service providers (phase 2), and concludes with group concept mapping co-design sessions (phase 3). This approach is appropriate for the aforementioned research objectives that require both measurement of relationships among psychosocial constructs and in-depth understanding of lived, professional, and organizational experiences [54]. The sequential design will allow Phase 1 survey findings to inform Phase 2 interview topics and Phase 3 co-design priorities. Recruitment for this study will begin on September 1^st^,2026 and will end on September 1^st^, 2028. All participants will provide informed consent prior to taking part in any study activity. For the survey, consent will be obtained electronically for participants completing the online survey, verbally for participants completing the survey by phone, and in writing for participants completing the paper version. For individual interviews and concept mapping/co-design sessions, written or electronic informed consent will be obtained before participation. Participants will be informed that participation is voluntary, that they may skip any question they do not wish to answer, and that they may withdraw from the study in accordance with the approved ethics protocol. results are expected. Survey results are expected in March 2027.

### Study setting

The study will be conducted in Canada on a sample of current and former refugee homestay hosts and service providers working in refugee-serving organizations, settlement, housing, community, or related organizations. Recruitment will be supported by national, provincial, and local refugee-serving organizations, community networks, hosting programs, faith groups, social media posts, and partner organizations.

### Community and stakeholder involvement

The study is designed as a research-to-policy and service practice project. Community partners and knowledge users will support recruitment, interpretation, co-design, and dissemination. Hosts and service providers will be engaged not only as participants but also as contributors to the co-design of practical outputs, including a host support toolkit, service provider guidelines, and policy recommendations. Phase 3 will provide a structured mechanism for participatory priority-setting through group concept mapping.

### Phase 1: National host survey

#### Participants and eligibility.

Phase 1 will recruit individuals who currently host or previously hosted a refugee or displaced person in a homestay arrangement in Canada. Participants will be eligible if they are 18 years of age or older, able to communicate in English, have current or prior refugee homestay hosting experience in Canada, and are able to provide informed consent. Individuals will be ineligible if they are under 18 years of age, have never hosted a refugee or displaced person in a homestay arrangement in Canada, are unable to provide informed consent, or are unable to participate in English.

### Recruitment and sample size

Recruitment will occur through partner organizations, refugee-serving organizations, hosting networks, community champions, and social media. The survey will first be pilot tested with approximately 15 hosts recruited via a collaborating community organization’s listserv. Following refinement, the survey will be launched nationally. The target sample size is at least 200 participants, consistent with common recommendations for structural equation modeling and sufficient power for most analyses [55,56].

### Data collection

The survey will collect data over approximately five months. Invitations will include a survey link through LimeSurvey and contain detailed study information. Follow-up reminders will be sent at two- and four-week intervals to maximize response rates [57]. The survey will be offered online, by phone, or in paper format. Participants will provide consent before beginning the survey. Survey completion is expected to take approximately 10 minutes. Participants will receive a CAD 20 e-transfer as a token of appreciation, with contact information collected separately from survey responses to maintain confidentiality.

### Measures

The survey will include a structured questionnaire developed for this study, combining validated self-report measures and study-specific items informed by hospitality studies, collective action theory, and prior refugee integration research [5,29,31,33,38,58–60]. Measures will include demographics such as age, gender, household income and provinces; hospitableness; readiness to assist; attitudes toward refugees; empathy toward refugees; satisfaction from hosting refugees; advocacy for hosting refugees; and open-ended questions about experiences, challenges, and successes. (***See* Table 1**).

**Table 1:** Phase 1 survey constructs and planned measures.

Construct	Description	Response format / source
Demographics	Age, gender, race or ethnicity, education, employment, household composition, years of hosting experience, and province or territory.	Study-specific items.
Hospitableness	Perceptions and enactments of reciprocity, care, welcome, and generosity.	Nine items adapted from Blain and Lashley [31], 5-point Likert scale.
Readiness to assist	Willingness to use personal resources, including time, property, attention, supplies, and integration support.	Items adapted from Korol and Bevelander [29], 5-point response options.
Attitudes toward refugees	Warmth and competence perceptions, including friendly, warm, trustworthy, tolerant, sincere, capable, efficient, organized, and skillful.	Nine items adapted from Knappert et al. [33] and Fiske [58], 5-point Likert scale.
Empathy toward refugees	Empathic responses to the experiences of refugees.	Eight items adapted from Davis [38], 5-point response options.
Satisfaction from hosting refugees	Overall evaluation of hosting experience.	Three items adapted from Oliver [59], 5-point Likert scale.
Advocacy for hosting refugees	Intentions to recommend, support, and promote refugee hosting.	Three items adapted from Wu and Chang [60], 5-point likelihood scale.
Open-ended questions	Nuanced views on hosting experiences, challenges, and successes.	Narrative responses analyzed thematically [61].

### Quantitative and qualitative analysis

**Fig 1:**
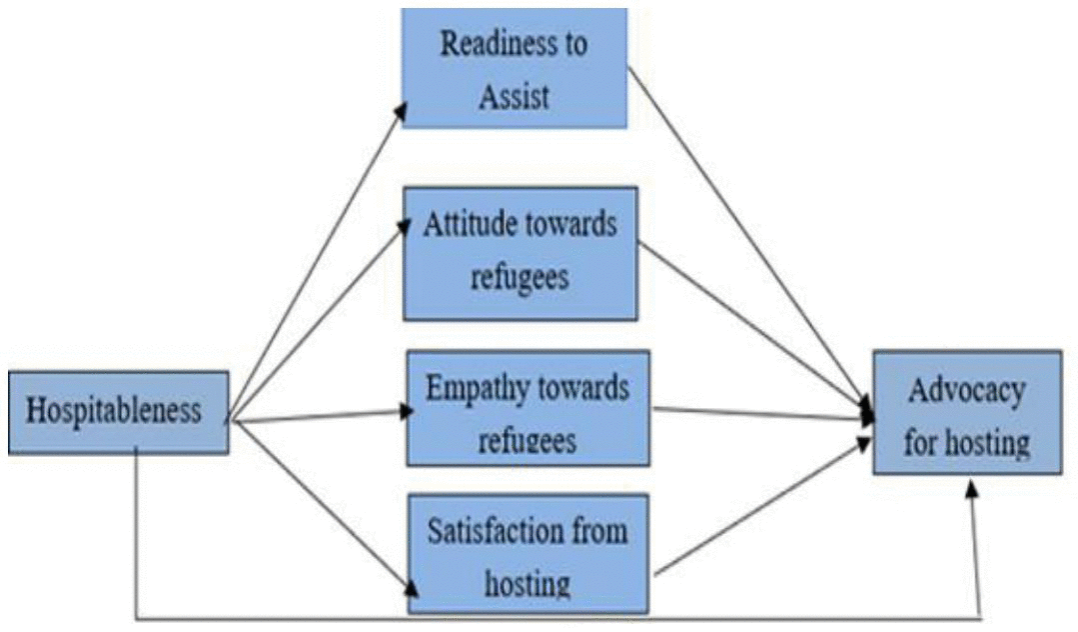
Conceptual model of the pathways linking hospitableness to advocacy for refugee hosting through readiness to assist, attitudes, empathy, and satisfaction with hosting.

Survey data will be analyzed using SPSS and SmartPLS version 4.0. Descriptive statistics will summarize participant characteristics and construct scores. Structural equation modeling will test the hypothesized mediation model examining relationships among hospitableness, attitudes, empathy, readiness to assist, satisfaction, and advocacy (***see*** Fig 1) Model fit will be evaluated using recommended indices, including chi-square, comparative fit index, root mean square error of approximation, and standardized root mean square residual [62–64]. Statistical significance will be set at .05. Open-ended survey responses will be analyzed thematically using NVivo 15, with hospitality studies and collective action theory used as theoretical lenses [61].

### Phase 2: Qualitative exploration with hosts and service providers

#### Design and participants.

Phase 2 will use qualitative descriptive methods to explore host and service provider experiences, needs, barriers, and recommendations [65]. The study will purposively recruit 20–25 hosts and service providers working in refugee-serving, migrant-serving, settlement, housing, community, or related organizations.

The target sample of 20–25 participants is consistent with qualitative descriptive studies of comparable scope, where data adequacy is determined by informational sufficiency rather than statistical power [61,65]. Recruitment will continue until thematic saturation is reached — the point at which additional interviews yield no new codes or themes relevant to the study objectives. Given the heterogeneity of participants (hosts and service providers across diverse regions, roles, and experiences), a larger range is anticipated to capture sufficient variation. Saturation will be assessed iteratively during analysis, with the option to recruit additional participants if new themes continue to emerge.

Individual semi-structured interviews with both hosts and service providers will be conducted by phone or virtually through Zoom. Host eligibility criteria will include being 18 years of age or older, currently or previously hosting a refugee or displaced person in Canada, having relevant hosting experience, ability to consent, and ability to participate in English. Service provider eligibility criteria will include being 18 years of age or older, working or having worked in a relevant refugee-serving organizations, settlement, housing, community, or related organization, ability to consent, and ability to participate in English.

### Data collection

Host interviews will explore motivations for hosting, meanings and practices of hospitableness, readiness to assist, attitudes and empathy toward refugees, satisfaction, challenges, access to supports, gendered and trauma-related dynamics, and recommendations for improvement. Service provider interviews will explore organizational roles, services provided to hosts and refugees, host needs and preparedness, trauma- and gender-responsive care, systemic barriers, coordination across services, and strategies for sustaining hosting. Interviews are expected to last approximately 45–60 minutes and will be conducted online through Zoom or by phone. All interviews will be audio recorded with consent and transcribed verbatim. Participants will receive a CAD 50 e-transfer following interview completion.

### Qualitative analysis

Qualitative data analysis will begin alongside data collection. Data will be analyzed inductively and deductively using NVivo 15 [61]. Inductive coding will identify themes emerging from participant narratives, while deductive coding will draw on sensitizing concepts from hospitality studies and collective action theory. The analysis will attend to intersections of trauma, gender, migration, informal and formal support, and host-refugee power dynamics. The process will include familiarization, coding, theme development, refinement, interpretation, collaborative transcript review, and participant validation through member checking where feasible [61,66]. Findings will inform Phase 3 co-design activities and development of tailored host support tools and training materials.

### Phase 3: Group concept mapping co-design sessions

#### Design and participants.

Phase 3 will use group concept mapping, a structured participatory approach that combines qualitative group processes with quantitative mapping techniques to develop and prioritize practical recommendations [67–70]. Approximately 20–25 participants from earlier phases, including hosts and service providers with diverse identities, roles, and locations, will be purposively recruited. Virtual sessions will be designed to enhance accessibility and participation across geographic regions and time zones.

### Co-design procedures

Four co-design sessions are planned. Session 1 will generate statements in response to the focus prompt, “What is needed to enhance, support, and sustain safe, inclusive, and effective refugee homestay hosting in Canada?” Participants will brainstorm needs, gaps, actions, and solutions. Session 2 will involve sorting statements into conceptually similar groups and rating each statement for importance and feasibility. Session 3 will review and interpret point maps and cluster maps, including whether clusters reflect participant experiences and priorities. Session 4 will translate clusters into action-oriented recommendations, including host-level tools, service-level coordination strategies, system-level policy recommendations, trauma- and gender-responsive safeguards, and sustainability strategies. Participants will receive a CAD 50 e-transfer for each session attended.

Co-design sessions will bring together hosts and service providers in shared participatory spaces which, akin to most forms of qualitative data collection and collaboration [71], introduces potential power imbalances that require active facilitation. Service providers occupy institutional roles and may hold greater structural authority or confidence in group settings, which could shape whose priorities are centered in the mapping and prioritization process. Facilitators will be attentive to these dynamics, and session design will include deliberate strategies to ensure host voices — particularly those from equity-deserving groups — are not marginalized within the co-design process.

### Concept mapping analysis

Data from sorting and rating activities will be analyzed using Groupwisdom. Multidimensional scaling will generate a point map based on item similarity [72], and hierarchical cluster analysis will group related ideas into meaningful clusters [73]. A stress value will be used to assess map fit, with values around 0.285 considered acceptable in group concept mapping [74]. Rating data will identify priorities by importance and feasibility. Transcripts and field notes will be analyzed thematically. Integrated findings will be used to develop practical outputs and prepare recommendations with partners.

### Integration of mixed-methods findings

Integration will occur at multiple points. Phase 1 quantitative findings will inform qualitative questioning in Phase 2, including areas requiring explanation or contextualization. Phase 2 themes will inform the statements and priorities used in Phase 3 concept mapping. Final integration will compare and synthesize statistical relationships, qualitative narratives, and co-designed priorities to produce evidence-informed recommendations. The integrated findings will be organized around host readiness, hospitableness and relationship-building, trauma- and gender-responsive care, service access and navigation, system and policy support, community mobilization, and sustainability of hosting.

### Ethical considerations

This study has been reviewed and approved by a University Research Ethics Board (REB# 2026−152). Participants will provide informed consent before participation. Survey participants will consent electronically, verbally, or in writing depending on survey mode. Interview and co-design participants will provide consent before recorded sessions. Participation will be voluntary, and participants may skip any question or withdraw according to the approved ethics protocol with relevant support resources will be provided when needed. Audio recordings will be deleted after transcription. De-identified quotations may be used in reports, presentations, and publications with participant consent.

### Data management and confidentiality

All research data will be managed according to institutional ethics requirements. Survey responses, interview transcripts, field notes, and concept mapping data will be stored securely on encrypted, password-protected university-approved systems. Contact information for incentives and research summaries will be stored separately from survey and interview data. Participant names will not appear in reports, presentations, or publications. Pseudonyms or participant codes will be used, and direct quotations will be de-identified. Data will be retained for five years after study completion for potential secondary data analysis and then securely deleted.

### Expected outcomes and knowledge mobilization

The study will generate empirical and co-designed evidence to support safe, inclusive, and sustainable refugee homestay hosting in Canada [75]. Expected outputs include a host support toolkit, service provider guidelines, recommendations for enhancing safety and trauma- and gender-responsive practices, policy strategies for organizations and decision-makers, peer-reviewed publications, conference presentations, community forums, executive summaries, and a short film documenting the co-design process and outcomes. Findings will be shared with participants who request a summary and will be deposited through a university public research repository, where appropriate.

## Discussion

This protocol describes a mixed-methods study designed to address a gap in refugee hosting scholarship and practice by centering Canadian hosts and service providers. The study is expected to advance understanding of how hospitableness, attitudes, empathy, readiness to assist, satisfaction, and advocacy interact in refugee homestay hosting [76]. It will also produce qualitative and co-designed evidence about the support required to sustain safe, trauma-informed and gender-responsive hosting and attentive to equity and belonging.

The study has several strengths. First, it integrates quantitative, qualitative, and participatory co-design methods, allowing the research team to examine both measurable psychosocial pathways and contextualized hosting experiences. A sequential rather than concurrent mixed-methods design was selected to allow each phase to systematically inform the next, ensuring that qualitative inquiry is grounded in quantitative findings and that co-design priorities reflect both. The trade-off is that survey items cannot be refined based on early interview insights, and the phases unfold over a longer timeline. This sequential logic was judged appropriate given the exploratory nature of host motivations in the Canadian context, where qualitative depth was needed to interpret, rather than simply parallel, the quantitative findings.

Second, it uses an interdisciplinary theoretical framework that recognizes hosting as both a domestic hospitality practice and a form of collective action [77]. This theoretical framework guided us in selecting our initial list of survey measures included in Table 1. Given that a few of these measures were derived from other contexts and thematic studies, the early pilot phase of survey data collection will provide an initial opportunity to empirically assess face validity and item clarity with Canadian hosts. We will subsequently revise the measure list where applicable.

Third, the involvement of community partners and hosts enhances the practical relevance of the study outputs. Finally, the use of group concept mapping provides a structured method for translating findings into prioritized action strategies. By studying the perspectives of all involved knowledge users, people with lived experience and stakeholders it can have immediate impact in the field.

Potential limitations include reliance on self-selected participants, possible underrepresentation of hosts who had negative experiences or disengaged from hosting networks, and the use of English-language participation criteria. We will, therefore, analyze and present our findings as representing the perspectives of relatively engaged hosts, and practice caution in generalizing conclusions to the broader population of Canadians who host or have hosted refugees.

Cross-sectional survey data will limit causal inference since mediation carries implicit temporal assumptions about the sequencing of effects that cross-sectional designs cannot confirm. Causal language will therefore be avoided in reporting Phase 1 findings, and relationships among hospitableness, attitudes, empathy, readiness to assist, satisfaction, and advocacy will be interpreted as associational. Further, the evolving nature of refugee hosting programs may influence recruitment and participant experiences during the study period.

Despite these limitations, the study is positioned to generate actionable policy implications for refugee homestay hosting in Canada. Findings on host engagement could inform whether Canada moves toward a more formalized national hosting framework, rather than continuing to rely on a fragmented network of local organizations. Evidence on motivational pathways linking hospitableness, empathy, and satisfaction could help organizations design more targeted recruitment and retention strategies, while findings on trauma and gender could support advocacy for minimum safeguards for refugee women — a tangible gap in current policy.
